# Upregulation of Potassium Voltage-Gated Channel Subfamily J Member 2 Levels in the Lungs of Patients with Idiopathic Pulmonary Fibrosis

**DOI:** 10.1155/2020/3406530

**Published:** 2020-02-25

**Authors:** Jong-Uk Lee, Hun Soo Chang, Chang An Jung, Ryun Hee Kim, Choon-Sik Park, Jong-Sook Park

**Affiliations:** ^1^Genome Research Center and Division of Allergy and Respiratory Medicine, Soonchunhyang University Bucheon Hospital, Bucheon, Republic of Korea; ^2^Department of Interdisciplinary Program in Biomedical Science Major, Soonchunhyang Graduate School, Bucheon, Republic of Korea

## Abstract

**Background:**

Fibroblast dysfunction is the main pathogenic mechanism underpinning idiopathic pulmonary fibrosis (IPF). Potassium voltage-gated channel subfamily J member 2 (KCNJ2) plays critical roles in the proliferation of myofibroblasts and in the development of cardiac fibrosis.

**Objectives:**

This study aimed to evaluate the role of KCNJ2 in IPF.

**Methods:**

*KCNJ2* mRNA expression was measured using real-time PCR in fibroblasts from IPF patients and normal controls (NCs). Protein concentrations were measured by ELISA in bronchoalveolar lavage (BAL) fluid obtained from NCs (*n* = 30), IPF (*n* = 30), IPF (*n* = 30), IPF (*n* = 30), IPF (*n* = 30), IPF (

**Results:**

*KCNJ2* mRNA expression was measured using real-time PCR in fibroblasts from IPF patients and normal controls (NCs). Protein concentrations were measured by ELISA in bronchoalveolar lavage (BAL) fluid obtained from NCs (*n* = 30), IPF (*n* = 30), IPF (*p* < 0.001). KCNJ2 protein levels in BAL fluid were significantly higher in IPF (6.587 [1.441–26.01] ng/mL) than in NCs (0.084 [0.00–0.260] ng/mL, *p* < 0.001). KCNJ2 protein levels in BAL fluid were significantly higher in IPF (6.587 [1.441–26.01] ng/mL) than in NCs (0.084 [0.00–0.260] ng/mL, *p* < 0.001). KCNJ2 protein levels in BAL fluid were significantly higher in IPF (6.587 [1.441–26.01] ng/mL) than in NCs (0.084 [0.00–0.260] ng/mL, *p* < 0.001). KCNJ2 protein levels in BAL fluid were significantly higher in IPF (6.587 [1.441–26.01] ng/mL) than in NCs (0.084 [0.00–0.260] ng/mL, *p* < 0.001). KCNJ2 protein levels in BAL fluid were significantly higher in IPF (6.587 [1.441–26.01] ng/mL) than in NCs (0.084 [0.00–0.260] ng/mL,

**Conclusion:**

KCNJ2 may participate in the development of IPF, and its protein level may be a candidate diagnostic and therapeutic molecule for IPF.

## 1. Introduction

Idiopathic interstitial pneumonia is a group of lung diseases of unknown etiology characterized by complex interactions among cells involved in inflammation and fibrosis. Idiopathic pulmonary fibrosis (IPF) is a well-known type of idiopathic interstitial pneumonia with a poor prognosis [1]. Although the disease course of IPF varies [2, 3], it is usually progressive in cases with irreversible pulmonary fibrosis. Several lines of evidence suggest that genetic and epigenetic mechanisms play roles in the development and prognosis of IPF [4]. Among the genetic factors, the expression of *MUC5B*, *SFTPC*, *SFTPA2*, *RTEL1*, *TERT*, and *hTR* is altered in the lungs of IPF patients compared with healthy subjects and those with other lung diseases [5]. Recently, global gene expression levels in lung tissues were compared to identify genes involved in the complex mechanism of IPF [6–9]. We also identified 178 of the total of 15,020 genes that were differentially expressed in lung fibroblasts from IPF patients and controls [10]. Among these genes, the mRNA expression of potassium voltage-gated channel subfamily J member 2 (KCNJ2) was 18.6-fold higher in IPF than control subjects.

As an inward rectifier potassium channel, KCNJ2 transports potassium ions (K^+^) more readily into the cell than out of the cell [11–14]. Because KCNJ2 is predominantly expressed in skeletal and cardiac muscle cells [15], this channel plays a physiological role in maintaining the resting potentials and controlling the excitability of cells [16, 17]. KCNJ2 knockdown decreased the Ca^2+^ influx and proliferative activity of atrial fibroblasts [18]. Exposure to low levels of K^+^, which induces hyperpolarization, enhanced the proliferation and survival of myofibroblasts; in contrast, exposure to elevated K^+^ caused depolarization [19]. Clinically, KCNJ2 is involved in the development of Andersen syndrome and atrial fibrillation. Additionally, this channel appears to be essential for maintaining the cellular functions of oligodendrocytes, astrocytes, epithelial cells, and fibroblasts [20–23]. Furthermore, phosphatidylinositol 4,5-bisphosphate, which maintains the KCNJ2 channel in the open state [16, 24, 25], is involved in cellular proliferation and angiogenesis in IPF [26]. Thus, KCNJ2 is expected to play a role in the development of IPF. However, KCNJ2 expression has not been evaluated in patients with IPF. Therefore, in the present study, we evaluated the relationships between KCNJ2 expression and the clinical characteristics of IPF by measuring KCNJ2 protein and mRNA levels in fibroblasts and bronchoalveolar lavage (BAL) fluid from normal controls (NC), patients with IPF, and patients with other interstitial lung diseases, including nonspecific interstitial pneumonia (NSIP), hypersensitivity pneumonitis (HP), and sarcoidosis.

## 2. Materials and Methods

We followed the methods provided by Lee et al. [10].

### 2.1. Study Subjects

Lung tissues and BAL fluids from patients with diffuse interstitial lung diseases were obtained from the biobank of Soonchunhyang University Hospital, Bucheon, Korea (schbc-biobank-2015-013) after approval of the study protocol by the Ethics Committee of Soonchunhyang University Hospital (SCHBC-IRB-2015-08-25). Informed written consent for study participation and sample donation was obtained from each subject. The diagnostic criteria for IPF, HP, NSIP, and sarcoidosis were based on an international consensus statement [1, 27–30]. All subjects were examined by a physician to obtain their medical history and undergo chest X-ray, high-resolution chest computed tomography (HRCT), and pulmonary function tests. None of the IPF patients had any evidence of underlying collagen vascular diseases according to laboratory tests and clinical symptoms. IPF was diagnosed by the presence of usual interstitial pneumonia (UIP) patterns in the pathological specimens (surgical IPF) and/or by HRCT in patients who did not undergo surgical lung biopsy (clinical IPF). Two pathologists examined each slide independently after being informed of the patients' sex, age, and HCRT results.

Pathological recognition of the NSIP pattern included two major aspects: (1) exclusion of other patterns of interstitial lung diseases and (2) categorization of the histological features according to the ATS/ERS 2002 classification [30, 31] and the modified histological definition of the NSIP pattern [32]. HP was diagnosed by the presence of clinical symptoms compatible with nonnecrotizing granulomatous bronchiolocentric pneumonitis [27]. The diagnosis of sarcoidosis was based on histological evidence of noncaseating granuloma and compatible clinical images [28, 29]. HP and sarcoidosis were diagnosed after excluding other diseases with similar histological profiles. Biopsy tissues were subjected to acid-fast bacilli and Gömöri methenamine silver staining to verify the absence of microorganisms and fungi. Serial DLCO and FVC were measured, and the annual rate of FVC decline was estimated as follows: (last FVC–baseline FVC)/baseline FVC/follow-up years. The NCs exhibited no respiratory symptoms, as determined by a screening questionnaire [32], and had a predicted FEV1 and FVC >80% and normal chest radiograms.

The total GAP score was calculated using the method suggested by Ley et al. [33]. The following four clinical variables were examined to determine the GAP score: sex (0 points for female, 1 point for male), age (0–2 points), FVC % (0–2 points), and DLCO % (0–3 points). We divided the patients into eight groups according to their GAP score: group 0 (*n* = 4), group 1 (*n* = 12), group 2 (*n* = 16), group 3 (*n* = 15), group 4 (*n* = 15), group 5 (*n* = 12), group 6 (*n* = 6), and group 7 (*n* = 4). DLCO was not measured in eight subjects with IPF and thus was excluded from the determination of their GAP scores.

### 2.2. Reverse Transcription Polymerase Chain Reaction (RT-PCR) and Real-Time PCR Detection of KCNJ2 mRNA of Cultured Fibroblasts

Lung fibroblasts were cultured from the surgical lung specimens of 14 IPF patients and 10 subjects who underwent surgery for stage I or II lung cancer, as described previously [34].

The extracted RNA was treated using the Turbo DNA-Free™ Kit (Ambion). The resulting total RNA (1 *μ*g) was suspended in diethylpyrocarbonate-treated water with 0.5 *μ*g 10 mM dNTPs and oligodeoxythymidine, heated at 65°C for 5 min, and then cooled on ice. PCR amplification was performed for 30 cycles (5 min at 94°C, 30 s at 94°C, 30 s at 60°C, and 30 s at 72°C) with a final extension at 72°C for 7 min. The PCR mixture (20 *μ*L) contained 1 *μ*g cDNA, 10 *μ*L 2 × Power SYBR Green PCR Master Mix (Applied Biosystems, Foster City, CA, USA), and 1 *μ*L 10 pmol forward and reverse primers. The following primer sequences were used: KCNJ2 (sense) 5′-CACTCCATGTCCCCATGCTC-3′ and (antisense) 5′-CCGCTACAGCATCGTCTCTT-3′; *β*-actin (sense) 5′-GGACTTCGAGCAAGAGATGG-3′ and (antisense) 5′-AGCACTGTGTTGGCGTACAG-3′. The PCR products were separated on a 1.0% agarose gel containing ethidium bromide in Tris-borate EDTA buffer at 100 V for 40 min and visualized under ultraviolet light. The KCNJ2 band intensities were normalized to those of *β*-actin. Real-time PCR was executed using the StepOne™ Real-Time PCR System (Applied Biosystems) in a two-step procedure: denaturation at 95°C for 15 s and 60°C for 1 min, followed by melting at 95°C for 15 s, 60°C for 1 min, and 95°C for 15 s. Expression levels were calculated using the 2^−ΔΔCT^ method [35] and expressed as the relative fold change after normalization to PPIA.

### 2.3. Enzyme-Linked Immunosorbent Assay (ELISA) of KCNJ2 in BAL Fluid

This study was performed using the KCNJ2 ELISA for the diagnosis in IPF between October 2015 and January 2018. The BAL procedure was performed in the lung segments exhibiting greatest disease involvement on HRCT under no immunosuppressive therapy, or in the right middle lobe of the NCs, as described previously [2, 36–38]. Differential cell counts were performed among 500 cells from BAL fluid placed on slides prepared using a cytocentrifuge and Diff-Quik staining. The total cell count was performed using a hemocytometer. Cells were removed from the supernatants by centrifugation (500 g, 5 min), and the supernatants were stored at−80°C. The KCNJ2 protein level was measured by ELISA (MyBioSource, San Diego, CA, USA) according to the manufacturer's recommendations. The lower limit of detection was 0.625 ng/mL, and values below this limit were set to 0. The interassay and intra-assay coefficients of variation were less than 15%.

### 2.4. Statistical Analysis

Receiver operating characteristic (ROC) analysis was performed, and the area under the ROC curve (AUC) and cutoff values were determined using MedCalc statistical software. Comparisons of the KCNJ2 level between groups were performed using the Kruskal–Wallis test and post hoc Mann–Whitney *U* test. Correlations between the KCNJ2 level and other parameters were analyzed using Spearman's correlation coefficient. The data are presented as medians with 25% and 75% quartiles for variables with a skewed distribution or as means ± standard error of the mean for variables with a normal distribution. The data were analyzed using SPSS v. 20.0.

## 3. Results

### 3.1. Clinical Characteristics of Study Groups

Fibroblast cultures were obtained from 14 IPF patients and 10 NCs who underwent lung surgery, and the clinical information and laboratory data of these subjects were summarized in our previous study [10]. BAL fluid samples were obtained from patients with IPF (*n* = 84), NSIP (*n* = 9), hypersensitivity pneumonitis (*n* = 8), and sarcoidosis (*n* = 10), and the clinical characteristics of these patients are summarized in Table 1. Patients with IPF had significantly higher macrophage, neutrophil, and eosinophil counts in the BAL fluid and lower FVC and FEV1 values than NCs (*p* < 0.05). The IPF group included 40 clinical and 44 surgical IPF patients, and their clinical characteristics were also summarized in our previous study [10].

### 3.2. KCNJ2 mRNA Levels in Cultured Fibroblasts

**Figure 1:**
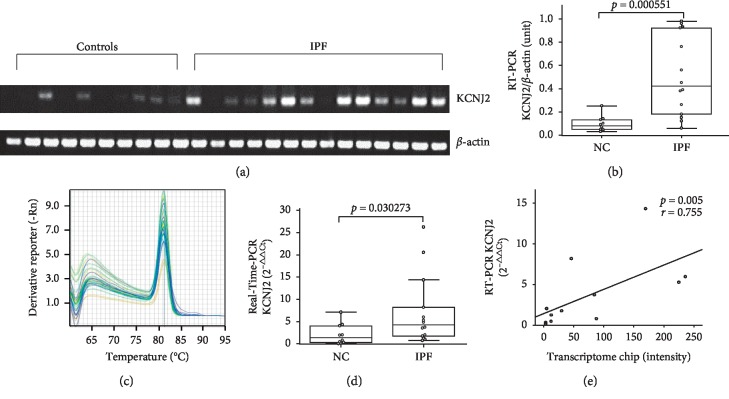
KCNJ2 mRNA levels in lung fibroblasts from 14 IPF patients and 10 NCs. (a) RT-PCR and (b) densitometric analysis of the KCNJ2 band intensities after normalization to those of *β*-actin. (c) Real-time PCR melt curves and (d) relative gene expression levels. (e) Correlations of the KCNJ2 mRNA levels (*n* = 12) between transcriptome chips [10] and real-time PCR. The data are presented as medians with 25% and 75% quartiles.

Fibroblasts from the lungs of the 14 IPF patients and 10 NCs were used to evaluate KCNJ2 levels. The KCNJ2 mRNA level in fibroblasts was two-fold higher in the IPF patients than in the NCs according to RT-PCR (*p* < 0.001; Figures 1(a) and 1(b)) and real-time PCR (*p*=0.03; Figures 1(c) and 1(d)). The KCNJ2 mRNA levels of our previously measured by transcriptomic analysis in the 12 subjects [10] showed a strong correlation with those determined by real-time PCR (*r* = 0.755, *p*=0.005; Figure 1(e)).

### 3.3. KCNJ2 Protein Levels in BAL Fluids

**Figure 2:**
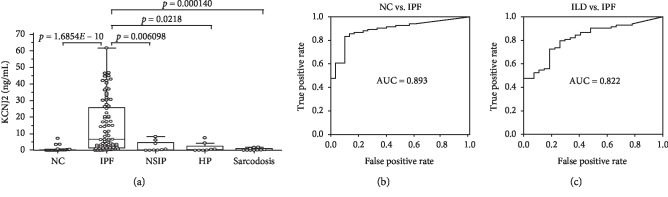
KCNJ2 protein concentrations in BAL fluids and ROC analysis. (a) KCNJ2 protein was detected in 18 of 30 NCs, 79 of 84 IPF patients, 7 of 9 NSIP patients, 5 of 8 HP patients, and 9 of 10 sarcoidosis patients. The data are presented as median values with 25% and 75% quartiles. (b) ROC curve of the KCNJ2 protein levels in IPF patients and NCs. A cut-off value of 0.636 ng/mL had an AUC of 0.893, a specificity of 90.0%, and a sensitivity of 83.3% for differentiating IPF patients from NCs. (c) ROC curve of the KCNJ2 protein level in patients with IPF and those with other interstitial lung diseases.

The protein levels of KCNJ2 in BAL fluids, measured by ELISA, were significantly higher in IPF patients (6.587 [1.441–26.01] ng/mL) than those in NCs (0.084 [0.000–0.260] ng/mL; *p* < 0.001), NSIP patients (0.301 [0.070–5.059] ng/mL; *p*=0.006), HP patients (0.365 [0.000–3.407] ng/mL; *p*=0.02), and sarcoidosis patients (0.170 [0.057–1.179] ng/mL; *p* < 0.001) (Figure 2(a)). The ROC curve showed a clear difference between the IPF patients and NCs (AUC = 0.893, Figure 2(b)) and between the IPF patients and groups with all other interstitial lung disease (*n* = 27) (AUC = 0.822, Figure 2(c)). The cutoff KCNJ2 level of 0.636 ng/mL determined from the ROC curve showed a 90.0% specificity and an 83.3% sensitivity for distinguishing between the NCs and IPF patients. The cutoff KCNJ2 level of 1.795 ng/mL exhibited an 81.48% specificity and a 72.62% sensitivity for distinguishing between the patients with IPF and those with other interstitial lung diseases.

### 3.4. Comparison of KCNJ2 Protein Levels according to IPF Clinical Characteristics

There were no differences in the KCNJ2 protein levels according to the GAP stage (stage I vs. II vs. III: 6.692 [1.244–26.59] ng/mL vs. 3.791 [1.676–17.10] ng/mL vs. 19.34 [3.142–42.10] ng/ml; Supplemental [Supplementary-material supplementary-material-1]), smoking status (SM vs. ES vs. NS: 7.765 [1.151–25.42] ng/mL vs. 6.375 [1.483–16.28] ng/mL vs. 6.692 [1.489–28.83] ng/mL; Supplemental [Supplementary-material supplementary-material-1]), sex (males vs. females: 6.375 [1.398–25.13] ng/mL vs. 6.692 [1.489–27.03] ng/mL; Supplemental [Supplementary-material supplementary-material-1]), or presence of arrhythmia (arrhythmia vs. no arrhythmia: 2.582 [0.596–3.232] ng/mL vs. 1.208 [0.239–3.975] ng/mL; Supplemental [Supplementary-material supplementary-material-1]).

## 4. Discussion

In this study, we demonstrated that KCNJ2 gene and protein expression was significantly increased in fibroblasts and BAL fluids, respectively, from patients with IPF compared with NCs and patients with other interstitial lung diseases. Additionally, the cutoff KCNJ2 protein level of 0.636 ng/mL exhibited 90.0% specificity and 83.3% sensitivity for diagnosing IPF. To the best of our knowledge, this is the first evidence suggesting that KCNJ2 expression is related to the development of IPF and that the KCNJ2 protein level may be a surrogate biomarker for differentially diagnosing IPF among other interstitial lung diseases. ROC curves showed a clear difference between the IPF patients and those with NSIP, HP, or sarcoidosis at a cutoff KCNJ2 protein level of 1.795 ng/mL.

We did not evaluate the cellular source of KCNJ2 production in BAL fluid, which is one of the limitations of the present study. Thus, it is uncertain whether fibroblasts or other cell types are the major source of KCNJ2 expression. K^+^ currents appear to be essential for the membrane functions of oligodendrocytes, astrocytes, and fibroblasts as well (18–21), even though KCNJ2 is expressed mainly in skeletal and cardiac muscle cells. Lung fibroblasts may express KCNJ2 upon activation, as in chronic inflammation and fibrosis in IPF, but this is still uncertain. Additionally, KCNJ2 is localized mainly near the plasma membrane [39]. Thus, KCNJ2 proteins were easily detected in BAL fluid in the present study.

KCNJ2 is associated with the gene ontology categories of G-protein-activated inward rectifier potassium channel activity and regulation of K^+^ flow. Although the ionic basis of the membrane potential of fibroblasts in IPF has not been fully elucidated, KCNJ2 is thought to affect the proliferation and membrane functions of these cells as well. This is supported by the finding that alterations in membrane potential by K^+^ currents modulated both the proliferation and morphology of cardiac myofibroblasts [19] in that exposure to low extracellular K^+^ concentrations induced hyperpolarization and enhanced myofibroblast proliferation and survival, whereas exposure to high concentrations enhanced myofibroblast contractility, according to collagen I gel deformation assays [19].

In noncardiac fibroblasts and myofibroblasts, K^+^ currents appear to be essential for maintaining the cellular functions of astrocytes [20], oligodendrocytes [23], and fibroblasts [22]. In each of these cell types, blocking K^+^ currents inhibited proliferation [21]. Depolarization resulted in enhanced ventricular myofibroblast contractility according to enhanced collagen I gel deformation upon an increase in K^+^ concentrations [19]. KCNJ2 expression mainly in cardiac muscle cells suggests a role for this protein in the development of Andersen syndrome and atrial fibrillation. Furthermore, atrial arrhythmias, including atrial fibrillation and atrial flutter, are the most common arrhythmias observed in patients with IPF [40]. The postulated mechanisms for arrhythmia development in these patients include the presence of hypoxia and chronic inflammation, elevated pulmonary pressure, and a risk of coronary artery disease [41]. However, genetic variants of *KCNJ2* may also contribute to the high incidence of atrial arrhythmias in IPF patients. Thus, we assessed the arrhythmia incidence in our 84 study subjects using electrocardiography (*n* = 80) and Holter monitoring (*n* = 5). Among them, six subjects had atrial fibrillation or flutter. There was no difference in the *KCNJ2* genotype frequency or BALF protein level between those with and those without atrial arrhythmia (Supplemental [Supplementary-material supplementary-material-1] and Supplemental [Supplementary-material supplementary-material-1]). Additionally, there was no significant difference in KCNJ2 expression according to sex or smoking status in the post hoc analysis (Supplemental Figures [Supplementary-material supplementary-material-1] and [Supplementary-material supplementary-material-1]). As a potential limitation of the present study, we did not evaluate the presence of hypoxia or pulmonary hypertension in the association analysis of atrial arrhythmias due to the small number of study subjects. In conclusion, KCNJ2 may be an indicator of the development of IPF and a surrogate marker for IPF differential diagnosis.

## Figures and Tables

**Table 1:** Clinical characteristics of the study subjects who underwent bronchoalveolar lavage.

Items	Normal controls	IPF	NSIP	HP	Sarcoidosis
No.	30	84	9	8	10
Age (year)	55 (35–72)	63.41 (59–75)	54.18 (39–70)†	44.58 (27–63)†	41.90 (27–68)†
Sex (male/female)	17/13	51/33	3/6	3/5	6/4
Smoke (NS/ES/SM/ND)	7/9/14	19/25/36	3/2/1/2	5/1/1/1	5/2/3/0
Follow-up duration (year)	ND	3.8 (1.7–6.5)	ND	ND	ND
Survival/Death	26/4	25/59	7/2	8/0	10/0
FVC (% pred.)	115.00 (106.25–132.25)	67.0 (52.0–80.00) ^*∗*^	62.00 (50.50–82.00) ^*∗*^	59.50 (54.50–81.50) ^*∗*^	86.50 (72.50–99.25) ^*∗*^†
FEV1 (% pred.)	104.00 (88.48–121.25)	89.50 (59.0–93.00) ^*∗*^	64.00 (56.50–94.00) ^*∗*^†	71.50 (58.00–92.00) ^*∗*^†	98.00 (80.50–106.25)
DLCO (% pred.)	86 (79–110)	57.00 (46.0–71.00)	62.00 (57.00–73.00)	68.50 (56.25–85.00)	89.00 (81.50–98.00)†
dFVC (%/year)	NA	−6.8 (–13.6–2.8)	NA	NA	NA
BAL total cell count (x104/mL)	3.46 ± 0.82	7.72 ± 2.42 ^*∗*^	18.56 ± 3.91 ^*∗*^	13.03 ± 3.78 ^*∗*^	9.13 ± 3.29 ^*∗*^
Macrophages (x104/mL)	3.02 ± 0.43	6.12 ± 1.78 ^*∗*^	12.91 ± 3.57 ^*∗*^	7.85 ± 2.38 ^*∗*^	7.26 ± 3.61 ^*∗*^
Neutrophils (x104/mL)	0.31 ± 0.047	1.87 ± 0.15 ^*∗*^	2.31 ± 1.42 ^*∗*^	3.42 ± 2.36 ^*∗*^	0.48 ± 0.18 ^*∗*^
Eosinophils (x104/mL)	0.02 ± 0.01	0.51 ± 0.08 ^*∗*^	0.72 ± 0.16 ^*∗*^	0.21 ± 0.18 ^*∗*^	0.12 ± 0.11
Lymphocytes (x104/mL)	0.02 ± 0.01	0.27 ± 0.15	2.32 ± 0.12 ^*∗*^†	2.40 ± 0.11 ^*∗*^†	2.44 ± 0.21 ^*∗*^†

IPF: Idiopathic pulmonary fibrosis; NSIP: Nonspecific interstitial fibrosis; HP: Hypersensitivity pneumonitis CS/ES/NS: current-smokers/ex-smokers/never-smokers; ND: not determined; dFVC (%): annual decline rate of FVC. Difference in patient characteristics and pulmonary function test, shown as median (IQR), among the controls, IPF, NSIP, HP, and sarcoidosis groups was calculated with Kruskal–Wallis analysis of variance and Mann–Whitney *U* test as post hoc test. BAL cell numbers, shown as mean ± standard error of the mean, among the five groups were compared using one-way ANOVA analysis of variance with Tukey's honestly significant difference test as post hoc test. Significance: compared with normal controls: ^*∗*^*P* < 0.05, compared with IPF: †*P* < 0.05.

## Data Availability

The data used to support the findings of this study are available from the corresponding author upon request.

## Abbreviations

IPF: Idiopathic pulmonary fibrosis
ECM: Extracellular matrix
BAL: Bronchoalveolar lavage
NC: Normal controls
NSIP: Nonspecific interstitial pneumonia
HP: Hypersensitivity pneumonitis
ROC: Receiver operating characteristic
IFN: Interferon.

## Supplementary Materials

Supplementary Materials Supplemental Figure 1: KCNJ2 protein concentrations in BAL fluids from IPF patients according to the GAP stage, smoking, and sex. Comparisons of KCNJ2 protein levels according to (a) the GAP stage, (b) smoking status, and (c) sex. Supplemental Figure 2: KCNJ2 protein concentrations in BAL fluids from IPF patients with or without atrial arrhythmias. Supplemental Table 1: comparison with genotype frequency of KCNJ2 in subjects with idiopathic pulmonary fibrosis with or without atrial arrhythmia.
