# Supplementary material for: Upregulation of Potassium Voltage-Gated Channel Subfamily J Member 2 Levels in the Lungs of Patients with Idiopathic Pulmonary Fibrosis
Source: Can Respir J. 2020 Feb 25;2020:3406530. doi: 10.1155/2020/3406530 (PMC7061125; doi:10.1155/2020/3406530)
Supplement: Supplementary Materials — Supplemental Figure 1: KCNJ2 protein concentrations in BAL fluids from IPF patients according to the GAP stage, smoking, and sex. Comparisons of KCNJ2 protein levels according to (a) the GAP stage, (b) smoking status, and (c) sex. Supplemental Figure 2: KCNJ2 protein concentrations in BAL fluids from IPF patients with or without atrial arrhythmias. Supplemental Table 1: comparison with genotype frequency of KCNJ2 in subjects with idiopathic pulmonary fibrosis with or without atrial arrhythmia. [file 3406530.f1.pdf]

Supplemental Figure 1

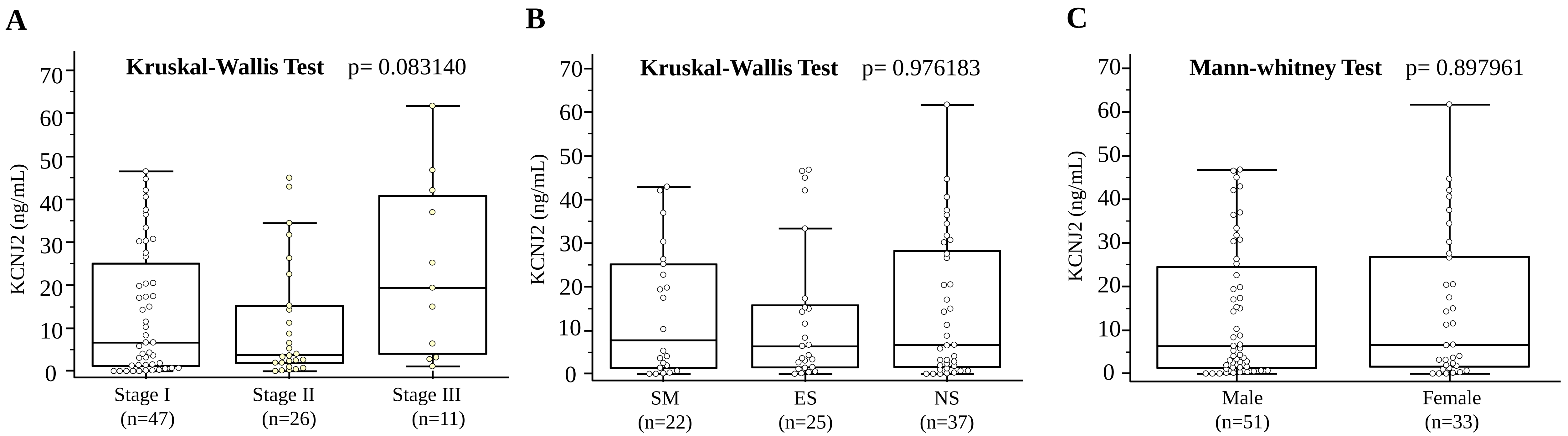

Supplemental Figure 2

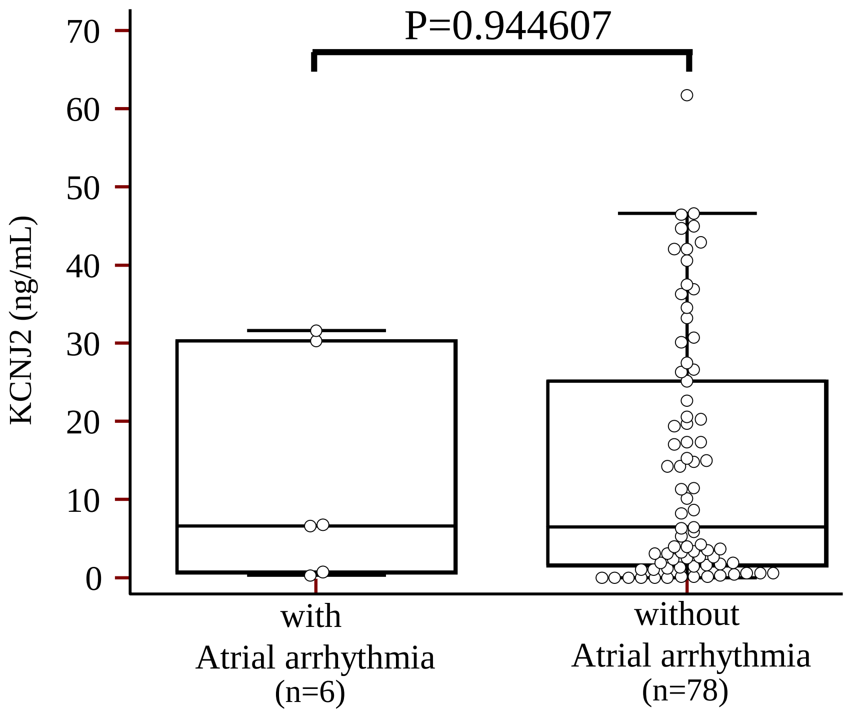

Supplemental Table 1. Comparison with genotype frequency of KCNJ2 in subjects with idiopathic pulmonary fibrosis with or without atrial arrhythmia

Chr	Gene	Exome-chip	Location	rs ID	Allele	Frequency	
						with Atrial arrhythmia	without Atrial arrhythmia
						Major/Hetero/Minor	Major/Hetero/Minor
17	KCNJ2	exm1349909	68171457	rs147750704	G>A	52/0/0	6/0/0
17	KCNJ3	exm1349927	68171796	rs141035459	G>A	52/0/0	6/0/0
17	KCNJ4	exm1349934	68172153	rs202067116	C>T	52/0/0	6/0/0
17	KCNJ5	exm1349948	68172409	rs141069645	A>G	52/0/0	6/0/0
